# A systematic approach to identify therapeutic effects of natural products based on human metabolite information

**DOI:** 10.1186/s12859-018-2196-0

**Published:** 2018-06-13

**Authors:** Kyungrin Noh, Sunyong Yoo, Doheon Lee

**Affiliations:** 1 Bio-Synergy Research Center, Daejeon, 34141 South Korea; 2 Department of Bio and Brain Engineering, Korea Advanced Institute of Science and Technology (KAIST), Daejeon, 34141 South Korea

**Keywords:** Natural product, Human metabolite, Medicinal compound, Similarity-based prediction, Data mining

## Abstract

**Background:**

Natural products have been widely investigated in the drug development field. Their traditional use cases as medicinal agents and their resemblance of our endogenous compounds show the possibility of new drug development. Many researchers have focused on identifying therapeutic effects of natural products, yet the resemblance of natural products and human metabolites has been rarely touched.

**Methods:**

We propose a novel method which predicts therapeutic effects of natural products based on their similarity with human metabolites. In this study, we compare the structure, target and phenotype similarities between natural products and human metabolites to capture molecular and phenotypic properties of both compounds. With the generated similarity features, we train support vector machine model to identify similar natural product and human metabolite pairs. The known functions of human metabolites are then mapped to the paired natural products to predict their therapeutic effects.

**Results:**

With our selected three feature sets, structure, target and phenotype similarities, our trained model successfully paired similar natural products and human metabolites. When applied to the natural product derived drugs, we could successfully identify their indications with high specificity and sensitivity. We further validated the found therapeutic effects of natural products with the literature evidence.

**Conclusions:**

These results suggest that our model can match natural products to similar human metabolites and provide possible therapeutic effects of natural products. By utilizing the similar human metabolite information, we expect to find new indications of natural products which could not be covered by previous in silico methods.

## Background

In drug development field, novel drug candidates are being continuously discovered, yet the approval rate of the new drug is decreasing compared to the budgets spent on the R&D [1]. Due to the large chemical space, it is laborious and difficult to find new therapeutic compounds. Even if we find new drug candidates, most of them are filtered out in various screening steps, such as bioactivity and toxicity. To solve this problem, many research has turned their attention to narrower chemical pools, like metabolites or natural products [2, 3].

When developing a new drug from a therapeutic compound, comparing its structure to known human endogenous metabolites is an essential step [4]. By comparing its structure, we may discover new positive effects or unwanted side effects caused by having a similar chemical structure of endogenous metabolites. Some of the drugs have been developed to mimic the structure of human metabolites [5]. Antihistamine is one straightforward example. By having the same binding site structure as the histamine with different residual structures, antihistamine antagonistically binds to histamine receptors and prevents histamine from initiating allergic reaction [6]. Other drugs like protirelin, which is a synthetic analogue of the thyrotropin-releasing hormone, or prednisone, which is an anti-inflammatory glucocorticoid derived from cortisone, have been synthesized to mimic human metabolites and bind to their target proteins [7].

As of now, DrugBank lists 2278 approved drugs, and Human Metabolome Database (HMDB) lists 29,266 recognized human metabolites [8, 9]. Among the 2278 approved drugs, 177 of them are exacted or derived compounds of human metabolites, according to the data in HMDB. While among 29,266 human metabolites, only 234 were developed into drugs. Such different proportions in drugs and human metabolites show the potential of new drug development from human metabolites.

In this study, natural products have been investigated to narrow down the chemical search space and find chemicals with high bioactivity. Natural products, which are secondary metabolites extracted from living organisms, have a distinct advantage in drug screening steps, for they are bioactive compounds in other organisms [2]. In previous work, principal component analysis (PCA) based chemical map was used to find natural products which are closely located to approved drugs in various feature spaces [10]. As a result, they found that natural products neighboring close to approved drugs show the same biological activity.

By definition, human metabolites also fall into the category of natural products in a broad sense. In the previous study, it has been shown that the natural products are more structurally similar to human metabolites than drugs are to human metabolites [11]. Also, the number of similar human metabolites of natural products were much larger than that of drugs. The result is relevant to the fact that natural products and human metabolites are both secondary metabolites in living organisms. By combining both human metabolite and natural product information, we could narrow down chemical search space with expected biological activities.

Here, we present a systematic method to discover new therapeutic compounds from natural products by using human metabolite information. Our main hypothesis is that natural products which are similar to human metabolites will have similar endogenous effects in our body. To define the similarity between natural products and human metabolites, we considered molecular and phenotypic properties by utilizing structure, target and phenotype similarities. By encompassing these three different aspects of molecular interaction, we expect to capture major features representing similarity between natural products and human metabolites. Using the three similarity features, we trained a support vector machine (SVM) model to match natural products to their similar human metabolites and assigned verified phenotype terms to each pair. From the result, we expect to find possible therapeutic effects of natural products, which may serve as new leads to drug developments.

## Methods

### Data collection

Human metabolite information was gathered from KEGG and HMDB [9, 12]. From KEGG, structure and target gene data were collected for 1051 human metabolites which have target information and are involved in human metabolic pathways. Associated phenotype terms of human metabolic pathways and known related phenotype terms of human metabolites were also gathered from KEGG and HMDB, respectively. As a result, 934 related phenotype terms were collected for 1033 human metabolites, with average 20 phenotypes per metabolites.

Natural product information was collected from TCM-ID, TCMID and CTD [13]. From TCM-ID and TCMID, we gathered the list of natural products, and the structure data and target gene information was downloaded for 18,583 natural products from CTD. From DrugBank, we selected 1873 approved drugs and collected their structure data and target gene information. Drug and human metabolite pairs were used when training an SVM model for selecting compounds similar to human metabolites.

Pathway data from Context-Oriented Directed Associations (CODA) was utilized to find affected phenotype terms of human metabolites and natural products [14]. CODA contains large endogenous information ranging from genes to organ levels and related phenotype terms ranging from symptom to disease, which enables us to simulate endogenous effects of compounds. From CODA, we extracted 1,007,771 relations among 20,046 genes and 7176 phenotypes.

### The similarity between natural products and human metabolites

**Fig. 1:**
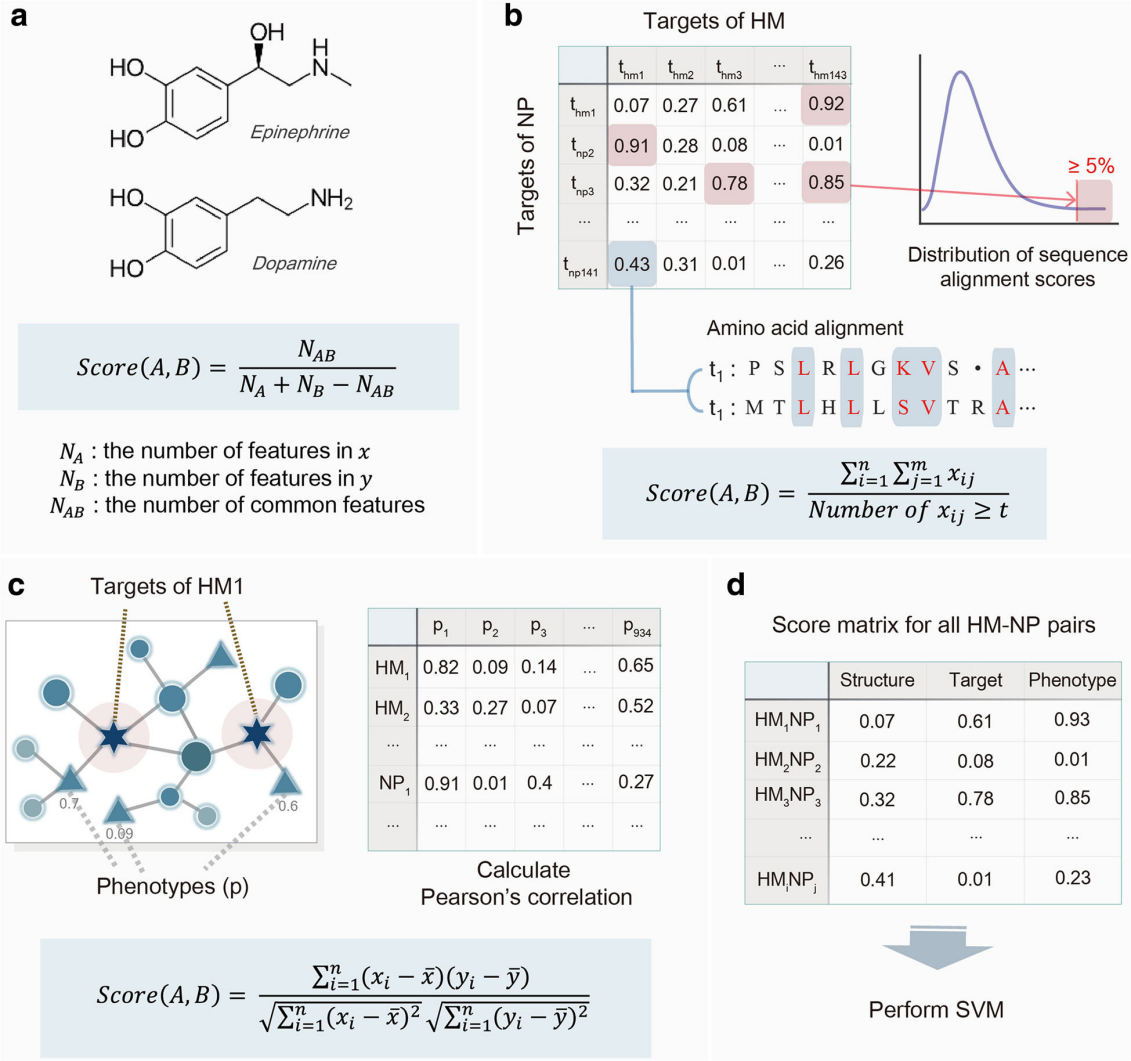
A systematic overview of the score matrix generation. Three scoring features, structure, target and phenotype, are utilized to generate score matrix of all possible human metabolite and natural product pairs. **a** Structural similarity is measured by Tanimoto coefficient. Molecular fingerprints of both human metabolite and natural product are compared using Tanimoto coefficient with bond sensitive, remove hydrogen and stereo filter settings in CDK. **b** Target similarity is measured by amino acid sequence similarity between two target proteins. Amino acid sequences of each target proteins of human metabolites and natural products are compared using Smith-Waterman algorithm. Among all possible target sequence similarity scores, scores over top 5% of the score distribution curve are selected and averaged to generate target similarity score of a human metabolite and natural product pair. **c** Phenotype similarity is measured by the random walk restart algorithm on the CODA network. For a human metabolite and natural product pair, vectors of all phenotype scores are compared by Pearson’s correlation. The absolute value of the Pearson’s correlation score is calculated for each human metabolite and natural product pairs. **d** Final score matrix is generated for all human metabolite and natural product pairs. The matrix is later utilized to train SVM model

To measure the similarity between natural products and human metabolites, we utilized three features, including structure, target and phenotype (Fig. 1). The structural similarity has been conventionally used to find drugs with similar biological activities [15]. In addition to the molecular similarity, we utilized target and related phenotype information to find similarities in binding proteins and overall phenotypic effects [16, 17]. The underlying hypothesis of our research is that natural products which are similar to certain human metabolites will have similar biological functions as the human metabolites. Therefore we measured the structural similarity with the whole sequence instead of the active domain to find compounds with similar phenotypes. With the same reasoning, when calculating the target similarity, we considered the whole amino acid sequence to predict biological functions.

#### Structural similarity

The structural similarity was measured by Tanimoto coefficient (Fig. 1a). With structure data of human metabolites and natural products in SMILES or MOL format, hashed fingerprints were generated using the Chemical Development Kit (CDK) to represent two-dimensional structures of compounds [18]. Then the Tanimoto score of a metabolite and natural product pair was calculated using bond sensitive, remove hydrogen and stereo filter settings in CDK.

$$ {S}_{A,B}=\frac{\left[{\sum}_{j=1}^n{x}_{jA}{x}_{jB}\right]}{\left[{\sum}_{j=1}^n{\left({x}_{jA}\right)}^2+{\sum}_{j=1}^n{\left({x}_{jB}\right)}^2-{\sum}_{j=1}^n{x}_{jA}{x}_{jB}\right]} $$

Here, *S*_*A*,*B*_ means the similarity between compound *A* and *B*, while *x*_*jA*_ means the *j*-th component of compound *A*.

#### Target similarity

Target similarity was measured by amino acid sequence similarity between two target proteins (Fig. 1b). For the sequence similarity measure, we used Smith-Waterman sequence alignment score, with the substitution matrix, BLOSUM-62. The sequence alignment score was further divided by the geometric mean of the self-alignment scores to be normalized, as suggested in Bleakley’s work [19]. From the calculated sequence alignment scores, we set the threshold value to cover top 5% of the distribution curve [20]. All target protein pairs of human metabolites and natural products were then filtered by the threshold value. Finally, the average sequence alignment score of the remaining target protein pairs was assigned to the human metabolite and natural product pair.

#### Phenotype similarity

Phenotype similarity was measured by the random walk with restart algorithm on the CODA network (Fig. 1c). CODA network contains large endogenous information ranging from gene to organ levels and related phenotype terms collected from various sources, which enable us to roughly simulate the endogenous effect of a compound. Random walk with restart algorithm propagates compound effects from initial nodes to their immediate neighbors for each step. As the step is repeated, compound effects will spread across the whole network, assigning probability values to each neighbor nodes [21].

$$ {p}_{s+1}=\left(1-r\right){M}^T{p}_s+{rp}_0 $$

Here, *p*_*0*_ is the initial probability vector, *p*_*s*_ is a probability vector in step *s*, and *M*^*T*^ is the transition matrix of the network. *r* represents the restart probability which is the probability of the random walker returning to its seed nodes. For this study, restart probability (*r*) was set as 0.7, and we determined that the random walker has reached the steady state when the difference between *p*_*s*_ and *p*_*s* + 1_ is less than 10^− 5^. After obtaining phenotype scores from the random walk algorithm, we used Pearson’s correlation to get phenotype similarity of all natural product and human metabolite pairs.

### Model generation

Using all three similarity measures, structure, target and phenotype similarity, a score matrix was generated for all natural product and human metabolite pairs (Fig. 1d). From this matrix, we expect to find similar human metabolites and map their related phenotype terms to each natural products. However, the gold standard set for the similar natural product and human metabolite pairs doesn’t exist. Therefore, we used drug and human metabolite pairs instead to train the SVM model. The similarity score of each drug and human metabolite pair was calculated by the same procedure used for natural product and human metabolite pairs. Among the 1873 drugs from DrugBank, 177 drugs, which are derived or designed from human metabolites, were chosen as the silver standard positive sets. The rest of the drug and human metabolite pairs were set as an unlabelled set and were randomly selected with the same number of positive sets to make a training set. We generated 100 random training sets and made SVM models for each set with 10-fold cross-validation.

To evaluate the performance of our trained models compared to the models trained with single similarity measure, each model was trained with single similarity features, and the area under the receiver operating characteristic (AUROC) scores were compared with the whole model. Also, to evaluate whether our model is highly dependent on chemical structure feature, separate models were trained with negative sets with high structure score (> 0.77) [22].

### Validation

To evaluate the performance of our SVM model, we used 10-fold cross-validation on drug and human metabolite pairs. The performance of the trained model on natural products was evaluated by the natural product derived drugs. Newman’s work provides lists of drugs which are derived from natural products [23]. Among them, we selected 391 drugs which are derived from or mimic of natural products. Indications of selected drugs were parsed from DrugBank, and we calculated the precision and recall of our method in predicting at least one of these indications. Finally, we found literature evidence to confirm the association between natural products and phenotypes. In PubMed abstracts, we manually counted co-occurrence of natural products and associated phenotypes, supported by the Jaccard Index and the Fisher’s exact test [24, 25].

## Results

### Correlation of similarity measures

Correlations among three similarity measures were computed to confirm whether some features are reliant on the others. For the measurement, Pearson’s correlation (*r*) was used for each feature scores of all positive set pairs. Target and phenotype features showed the highest correlation (*r* = 0.564) compared to structure and target (*r* = 0.452) or structure and phenotype (*r* = 0.531) features. Both target and phenotype features are generated from the target gene information of natural products and human metabolites, and it is reflected in the correlation. However, the overall correlation is low, showing that our model is not dependent on any single feature.

### Performance evaluation

**Fig. 2:**
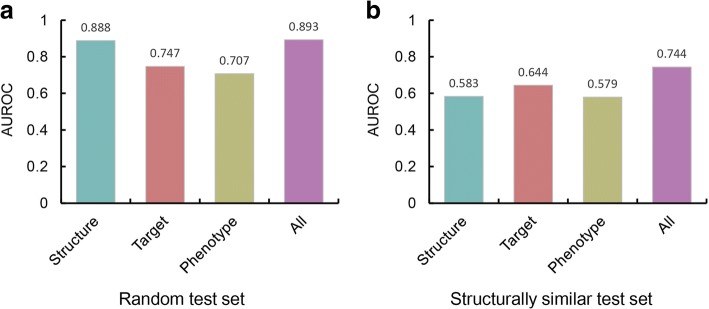
AUROC value of models generated from different feature sets. SVM models are trained with single features and compared with the model trained with the whole feature sets. **a** For random test set, SVM model trained only with structure feature shows as high AUROC as the model trained with the whole feature sets. **b** For structurally similar test set (Tanimoto score ≥ 0.77), all features contribute to improve the overall performance

We computed AUROC score of our trained SVM model and compared with the other models trained with a single feature. The test set was generated by randomly selecting negative sets to match the ratio to the positive sets. As a result, the model trained with all features showed the highest performance (AUROC = 0.893) compared to the models trained only with structure (AUROC = 0.888), target (AUROC = 0.747) and phenotype (AUROC = 0.707) features (Fig. 2a). Among all features, the structure feature had the highest score, reflecting its dominant role in our model. It was an expected result since we used drugs derived from human metabolites as a positive set in the training of the SVM model, where most of them had high structural similarity. To check the influence of the other features on our model, we tested our models on the test set with high structure similarities which was constructed by selecting drug and human metabolite pairs satisfying Tanimoto scores higher than 0.77. Based on previous studies, Tanimoto score of 0.77 was set as a threshold value for judging structural similarity [11]. By filtering the structurally similar drug-metabolite pairs, we decreased the effect of structure feature and trained our model. The model trained with all features showed the highest performance (AUROC = 0.744) while the models trained with other features, structure (AUROC = 0.583), target (AUROC = 0.644) and phenotype (AUROC = 0.579), showed similar performance (Fig. 2b). From the result, we could confirm that although the structural similarity is the dominant feature in our model, the other features, target and phenotype similarities, are also important in the model.

**Fig. 3:**
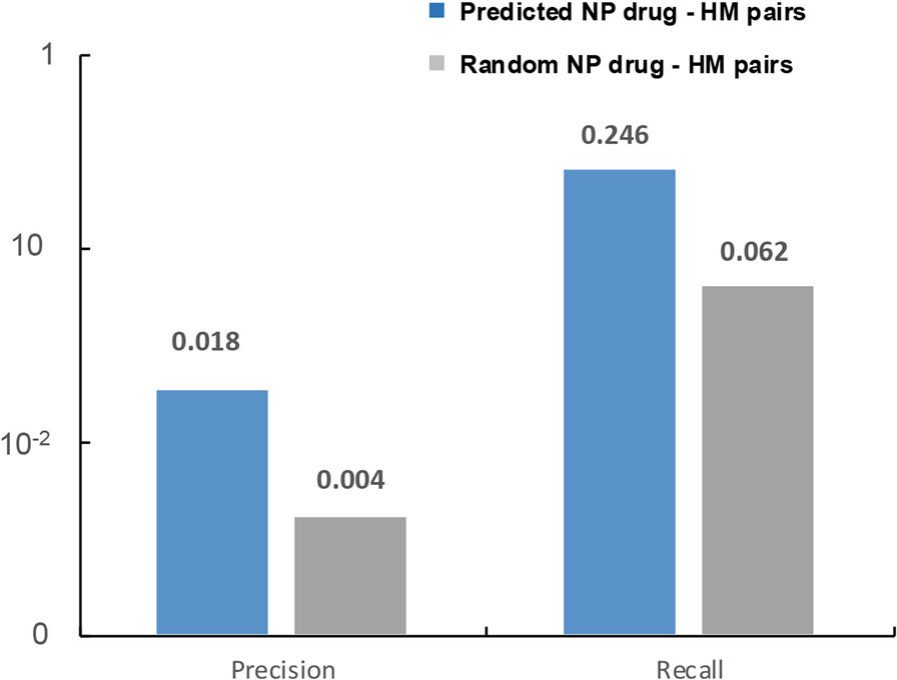
Performance of predicting natural product derived drugs. For all natural product derived drugs listed in the DrugBank, we evaluated whether our model can predict at least one indication of them. The blue bar represents natural product and human metabolite pairs which are predicted by our method as being similar, and grey bar represents the randomly matched natural product and human metabolite pairs. The predicted pairs generally show four times higher performance than the random pairs

In this study, we found similar human metabolites and mapped their related phenotype terms to each natural products. To evaluate the performance of phenotype prediction, precision and recall values were calculated for the verified phenotype terms of natural products. Natural products which are being used as drugs were selected as a positive set. For the indications listed in the DrugBank for each natural product drug, we evaluated whether our model can predict at least one indication of them. In Fig. 3, blue bar represents natural product and human metabolite pairs which are predicted by our method as being similar, and grey bar represents the randomly matched natural product and human metabolite pairs. The predicted pairs (precision = 0.018, recall = 0.246) generally show four times higher performance than the random pairs (precision = 0.004, recall = 0.062). Since the number of negative sets is 100-folds larger than the number of positive sets, the precision was very low. However, the comparison with the random samples shows that our result holds statistical importance.

### Literature validation for natural products and associated phenotype

The natural product and human metabolite pairs and resulting associated phenotype terms were validated by external literature (Table 1). Total 1930 natural product and human metabolite pairs, composed of 442 natural products and 365 human metabolites, were predicted by our method. Using PubMed abstracts, we counted co-occurrence (*n*_*c*_) of each pair and calculated Jaccard Index (JI) and Fisher’s exact test score (*n*_*f*_), compared to the random pairs. To check the difference from the random set, we performed Mann-Whitney U test and calculated corresponding *p*-values [26]. A *p*-value lower than 0.05 was considered statistically significant. The average number of co-occurrence was ten times higher in our result pairs (*n*_*c*_ = 45.09, *p*-value = 0.0021) than in random pairs (*n*_*c*_ = 3.77). Also, the average Jaccard index score and Fisher’s exact test score were comparably higher in our result pairs (JI = 1.87 × 10^− 3^, *n*_*f*_ = 266, *p*-value = 0.0001) compared to random pairs (JI = 8.21 × 10^− 5^, *n*_*f*_ = 38). We further confirmed the association between natural products and phenotypes paired by similar human metabolites. Among 4168 natural product and phenotype pairs, the average co-occurrence of a pair (*n*_*c*_ = 3.78, *p*-value = 0.016) was higher than the random pairs (*n*_*c*_ = 1.47). The average Jaccard index score as similar in our result pairs (JI =1.13 × 10^− 3^, *p*-value = 0.0155) and random pairs (JI = 1.16 × 10^− 4^). Finally, the Fisher’s exact test scores of our result pairs (*n*_*f*_ = 52, *p*-value = 0.0011) were remarkably higher than the random pairs (*n*_*f*_ = 6). These results show that our method can successfully associate phenotype terms to natural products using human metabolite information.

**Table 1:** Literature validation for natural products, human metabolites and phenotype association

	Natural product-human metabolite		Natural product – phenotype	
	Our method	Random	Our method	Random
Co-occurrence	45.09	3.77	3.78	1.47
Jaccard index	1.87 × 10^−3^	8.21 × 10^− 5^	1.13 × 10^− 3^	1.16 × 10^− 4^
Fisher’s exact test^a^	266	38	52	6

^a^The number of significant associations satisfying the Fisher’s exact test *p*-value threshold (*p*-value < 0.001)

## Discussion

Currently, our method only covers three broad features; structure, target sequence and overall phenotype correlation. However, there are still many detailed features which can be used to measure similarities between natural products and human metabolites. For further improvement, other various factors may be included, and the overall performance should be measured to capture the most relevant feature sets. With further improvements, we believe that our method can serve as a new search tool to find drug candidates from natural products.

## Conclusions

Identifying therapeutic compounds with high bioactivity is an essential step in drug discovery. Due to the large chemical space, many researchers have turned their attention to natural products, which has strength in high bioactivity and traditional medicinal use cases. Most of the research has focused on chemical similarities or literature evidence to find therapeutic compounds from natural products. Here, we propose a different approach based on human metabolite similarity. Using structure, target and phenotype similarities, our method finds similar human metabolites and their associated phenotypes for natural products, introducing a new method of finding therapeutic compound candidates for drug development.

By utilizing human metabolite information, we can find bioactive compounds with therapeutic effects, unhandled in the previous literature based or chemical similarity-based methods. Similarity to human metabolites were calculated by considering molecular and phenotypic properties of natural products. Using structure, target and phenotype features, our method showed the highest performance (AUROC = 0.893) compared to the other methods trained with a single feature. When applied to the natural product derived drugs, our method could successfully identify indications of the drugs with high precision. These results support that similarity to human metabolites can serve as a new paradigm of natural product research, aside from literature or chemical similarity-based methods. We further found literature evidence for the association between natural products and phenotypes by manual curation. From the result, we could find that many of the phenotype terms mapped to natural products have been clinically researched in many studies.

## Abbreviations

AUROC: Area under the receiver operating characteristic
CDK: Chemical Development Kit
CODA: Context-Oriented Directed Associations
CTD: Comparative Toxicogenomics Database
HMDB: Human Metabolome Database
KEGG: Kyoto Encyclopedia of Genes and Genomes
PCA: Principal component analysis
SVM: Support vector machine
